# Self-directed learning readiness of Indian medical students: a mixed method study

**DOI:** 10.1186/s12909-018-1244-9

**Published:** 2018-06-08

**Authors:** Kalyani Premkumar, Elizabeth Vinod, Solomon Sathishkumar, Anna B. Pulimood, Valerie Umaefulam, P. Prasanna Samuel, Tara A. John

**Affiliations:** 1 HSC E-wing 3226 Department of Community Health and Epidemiology, College of Medicine, University of Saskatchewan, Saskatoon, SK Canada; 2 Christian Medical College, Vellore, India

**Keywords:** Self-directed learning, Medical education, SDLRS score, Curriculum, Culture

## Abstract

**Background:**

Self-directed learning (SDL) is defined as learning on one’s own initiative, with the learner having primary responsibility for planning, implementing, and evaluating the effort. Medical education institutions promote SDL, since physicians need to be self-directed learners to maintain lifelong learning in the ever-changing world of medicine and to obtain essential knowledge for professional growth. The purpose of the study was to measure the self-directed learning readiness of medical students across the training years, to determine the perceptions of students and faculty on factors that promote and deter SDL and to identify the role of culture and curriculum on SDL at the Christian Medical College, Vellore, India.

**Methods:**

Guglielmino’s SDL Readiness Scale (SDLRS) was administered in 2015 to six student cohorts (452 students) at admission, end of 1st, 2nd, 3rd and 4th year of training, and at the beginning of internship in the undergraduate medicine (MBBS) program. Analysis of variance (ANOVA) was used to compare SDL scores between years of training. 5 student focus groups and 7 interviews with instructors captured perceptions of self-direction. Transcripts were coded and analyzed thematically.

**Results:**

The overall mean SDLRS score was 212.91. There was no significant effect of gender and age on SDLR scores. There was a significant drop in SDLRS scores on comparing students at admission with students at subsequent years of training. Qualitative analysis showed the prominent role of culture and curriculum on SDL readiness.

**Conclusions:**

Given the importance of SDL in medicine, the current curriculum may require an increase in learning activities that promote SDL. Strategies to change the learning environment that facilitates SDL have to be considered.

**Electronic supplementary material:**

The online version of this article (10.1186/s12909-018-1244-9) contains supplementary material, which is available to authorized users.

## Background

Self-directed learning (SDL) is a vital educational principle in higher education that has been promoted by various institutions due to its value in developing professionals to become lifelong learners. Medical education systems worldwide have embraced SDL so that medical students gain SDL skills to continuously equip themselves with relevant knowledge and skills in the ever evolving world of medicine. SDL is generally defined as learning on one’s own initiative, with the learner having primary responsibility for planning, implementing, and evaluating the effort [1]. In medical education, SDL is the process in which medical students take the initiative, with or without the help of others (e.g. instructors and colleagues), determine their learning needs, set learning goals, identify resources for learning, choose and implement learning strategies to acquire knowledge and finally evaluate learning outcomes [2]. Hence, the medical student is responsible for his or her own learning. SDL readiness (SDLR) is the extent to which the student has the ability, attitude and personal characteristics appropriate for SDL [3].

In a constantly changing environment, SDL is essential to enable medical students to develop independent learning skills, increased responsibility, assertiveness and accountability which are key attributes to a medical professional’s career. Medical educators similarly seek to adopt SDL with the primary aim of producing learners who can manage their own learning in their careers and have a continuous quest for knowledge through critical thinking that will enhance retention and recall of information to promote better decision making [4].

Thus, health professionals need to be self-directed so as to increase independence, self-confidence in practice, motivation, self-discipline and goal orientation due to information explosion and the continuously evolving medical knowledge during their careers [5].

### Medical education in India

The Medical Council of India (MCI) has set uniform standards for medical education in India. In 2011, the MCI modified the curriculum and provided directions for teaching and learning methodology in medical education – Vision 2015, so that Indian medical graduates can be lifelong learners [6]. Thus, the curriculum followed by medical institutions, seek the development of SDL readiness in students [7] as promoted by MCI. However, monitoring changes and quality of education is a huge challenge given that India has the largest number of medical colleges globally (*n* = 460).

### Christian medical college, Vellore, curriculum and SDL

The Christian Medical College (CMC) is one of the leading medical institutions in India, committed to excellence in education, patient care and research. It provides quality primary, secondary, tertiary and quaternary patient care services, with a special concern for the poor and the marginalized. Hence, yearly admission into this medical college is rigorous and highly competitive [8].

The medical program at CMC, Vellore, is a four and a half years program followed by one year of internship. The program is organized with a mix of subject-based, community-based, competency-based and problem-based curricular components. There are several opportunities for SDL within the curriculum which include the Integrated Learning Programs [9], early clinical exposures [10], clerkship programs, laboratory practicals, chart discussions, tutorials, student seminars, e-learning, projects in the community, bedside clinics, research projects, prize examinations and secondary hospital programs.

### Culture and education

Culture is the set of meanings that a group in a time and place come to adopt or develop, and culture can also be regarded as how people think and address various situations/experiences which may be based on individualism, collectivism, or honor [11]. Culture is a dominant controlling factor that impacts one’s way of learning and communication in an academic setting [12]. In general, students learn attitudes, norms, practices, and beliefs even before being exposed to formal education. Values such as unity, tolerance, obedience and respectfulness are instilled from a young age via culture and customs and these influence the way students learn. Thus, students share ideas and practice their cultural values even in an academic environment because it is a part of their life.

India is rich in culture and it plays a major role in the process of learning. The instructors also bring into classrooms, beliefs based on their own experiences [13]. Parents play a key role in their children’s education and the social environment in which families live influences their involvement [14].

Recent trends in medical education have shown an increase in the adoption of student-centred methods such as problem-based learning (PBL) that emphasize SDL. The acceptability of such methods is not universal and shows variation across different cultures and countries [15].

Cultural factors that impede SDL in medical students across cultural groups differ. Frambach et al. [15] found that uncertainty and tradition were principle restraints in Middle Eastern students’ SDL, whereas a dependence on hierarchical sources rather than oneself was challenging to SDL in Asian students. The pressure of achievement was high in non-Western students. These factors had minimal influence on students in Western countries [15].

It was noted, however, that once introduced, students grew accustomed to newer methods of education; and acceptability as well as skills in SDL increased across different cultures despite the various challenges in each setting [15].The impact of culture is seen not only in development of readiness to SDL but also in communication and learning strategies adopted [16].

The curriculum at CMC has various components that promote SDL such as the Integrated Learning Program. It has not been studied whether the students are indeed self-directed and whether the curriculum and culture promote or deter self-direction. Given that SDL is an important skill required for lifelong learning, it is vital for this question to be investigated in order to promote SDL among Indian medical students.

### Purpose of study

The aims of the study were to:

1. Measure the SDLR of medical students at admission and students at different stages of medical training.
2. Determine the perceptions of students and faculty regarding SDL and factors that promote and deter SDL.
3. Identify the role of culture and curriculum on SDL.

## Method

This is a cross-sectional study that investigated the readiness for SDL among undergraduate medical students at CMC, Vellore. Institutional Review Board approval was obtained prior to the start of the study. As a result of the importance of this study to the curricular mandate of CMC, the researchers invited all students in the medical program to participate in the study and 453 students participated. Participating students and faculty gave informed consent.

### Conceptual framework and the instrument

The Guglielmino’s self-directed learning readiness scale (SDLRS) is a self-scoring instrument designed to assess attributes supportive of SDL based on individual personality characteristics, values, attitudes and skills [17]. The instrument consists of a self-report questionnaire of 58 questions and is a common instrument used for assessing SDL readiness [18]. It measures eight factors including creativity, love of learning, initiative and independence in learning, openness to learning opportunities, informed acceptance of responsibility to one’s own learning, self-concept as an effective learner, ability to use basic study and problem-solving skills and positive orientation to the future.

The SDLRS utilizes a five-point Likert scale (1 = Almost never true of me; I hardly ever feel this way; 5 = Almost always true of me; there are very few times when I don’t feel this way). Examples of the Likert statements include ‘I’m looking forward to learning as long as I’m living‘, ‘I know what I want to learn‘, ‘when I see something that I don’t understand, I stay away from it‘, ‘if there is something I want to learn, I can figure out a way to learn it’. The scores range from 58 to 290, with an average adult score of 214 (±25.59) [17]. The scores are interpreted as 58 to 201 (below-average SDL readiness), 202 to 226 (average SDL readiness), and 227 to 290 (above-average readiness). The scores show that individuals present SDL readiness state with the ability to improve with appropriate educational interventions (Guglielmino and Associates). The instrument has a test–retest reliability of 0.829 and 0.79, a Pearson split-half reliability estimate of 0.94, and a Cronbach alpha reliability coefficient of 0.87 [1]. As a result of the extensive study of this instrument and usage in multiple studies, it is considered to be accurate and useful for measuring SDL readiness.

### Participants

Prior to administration of the SDLRS (described above), the study’s objective was explained, and the researchers followed the protocol for administration, as provided by the SDLRS developers to the participants. The instrument was administered to six cohorts of students enrolled in the MBBS program (at admission, at the end of 1st, 2nd, 3rd and 4th year of training, and at the beginning of internship) (Additinal file 1). A total of 453 students participated in the study but one student data had to be discarded because this participant left many questions unanswered. The SDL scores obtained for 452 students were analyzed.

The researchers invited faculty in the medical school to participate in semi- structured interviews. Seven members of the faculty took part in the interviews. In addition, students from each year of training participated in focus group discussions. There were five focus group discussions conducted with each cohort of students. The focus group discussions and interviews were held to gain a better understanding of the participants’ definition of SDL, factors facilitating and deterring SDL as well as perceptions on culture and curriculum affecting SDL. All focus groups and interviews were recorded and transcribed. The transcriptions were analyzed by two of the researchers for common themes.

### Data analysis

SPSS (Statistical Package for Social Sciences–Version 19, Chicago, Illinois) was used for analysis. Descriptive statistical methods were used to summarize all study variables. Analysis of variance (ANOVA) was used to test the difference in mean SDLRS scores between years of training. The dependent variable was SDLRS scores and years of training (defined categorically as 0 = at admission, 1 = end of 1st year, 2 = end of 2nd year, 3 = end of 3rd year,4 = end of 4th year of training, 5 = Beginning of Internship) was used as independent variable. Post-hoc analysis was performed using Bonferroni correction. Results were presented as means with 95% confidence intervals. All results were analyzed using α = 0.05.

The recorded interviews and focus group discussions were transcribed (Additional file 1). The primary researcher and a content and qualitative expert independently examined transcripts for common themes. Data was analyzed via thematic analysis and repeated discussions were held until the coders reached a consensus.

## Results

There were more female students as compared to males (Table 1).

**Table 1:** Distribution of Participants

SEX	Freq.	Percent
Male	174	38.63
Female	258	56.95
Missing	20	4.42
Total	452	

Missing = Gender data not entered

### Measurement of SDL

**Fig. 1:**
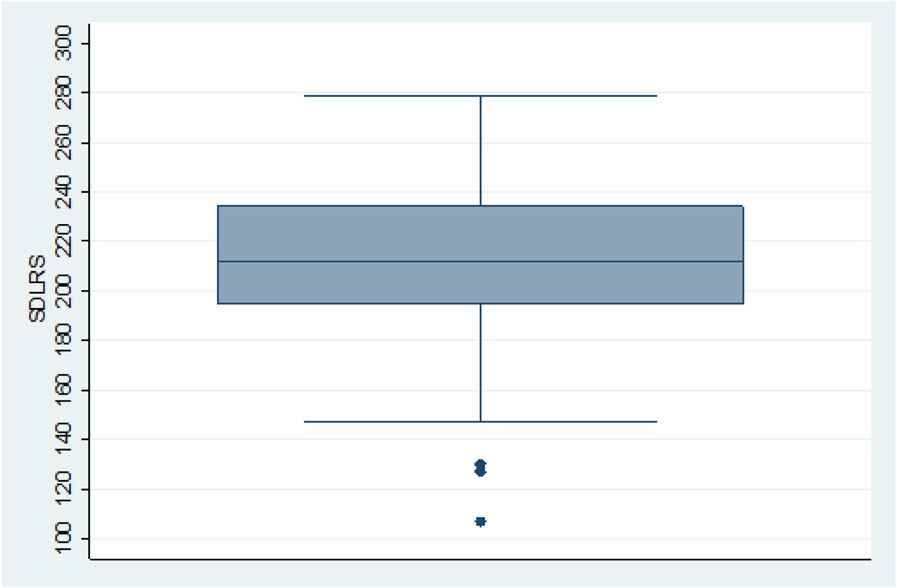
Box plot showing the mean SDLR score of all the medical students

**Fig. 2:**
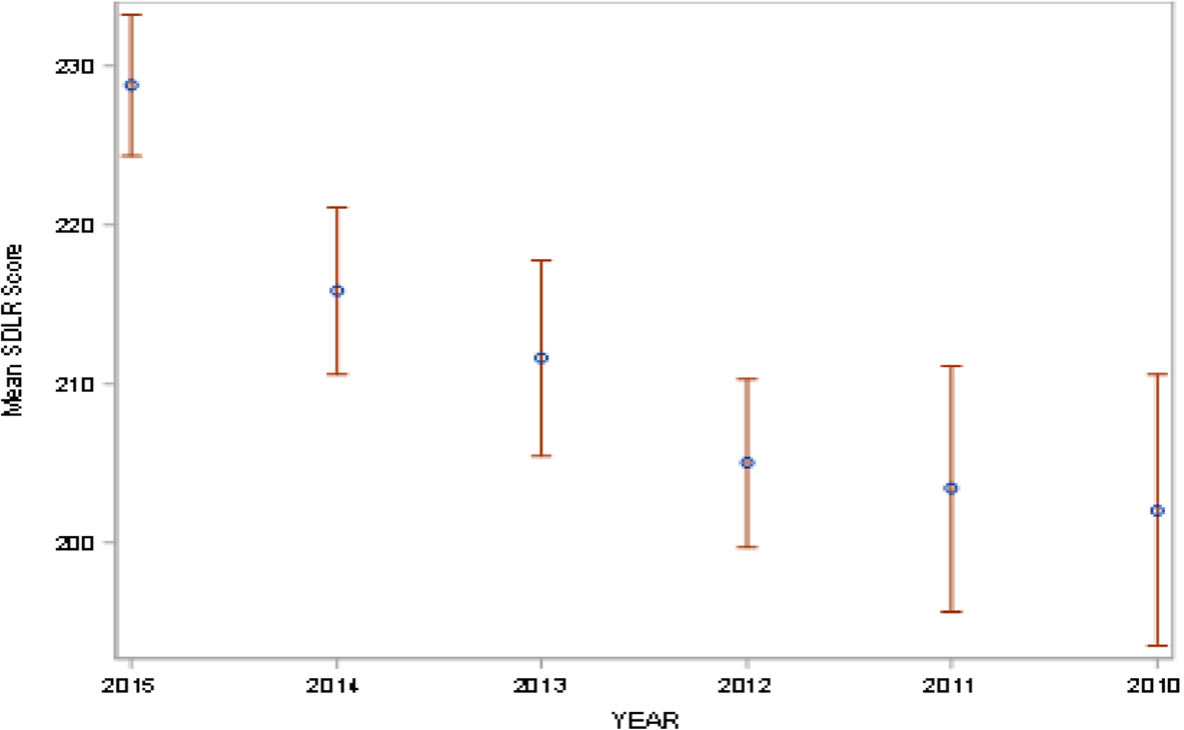
Mean SDLR scores with 95% confidence intervals at admission, end of 1st, 2nd, 3rd and 4th year of training, and at the beginning of Internship (Batch of 2015, 2014, 2013, 2012, 2011 & 2010 respectively based on year of admission)

**Fig. 3:**
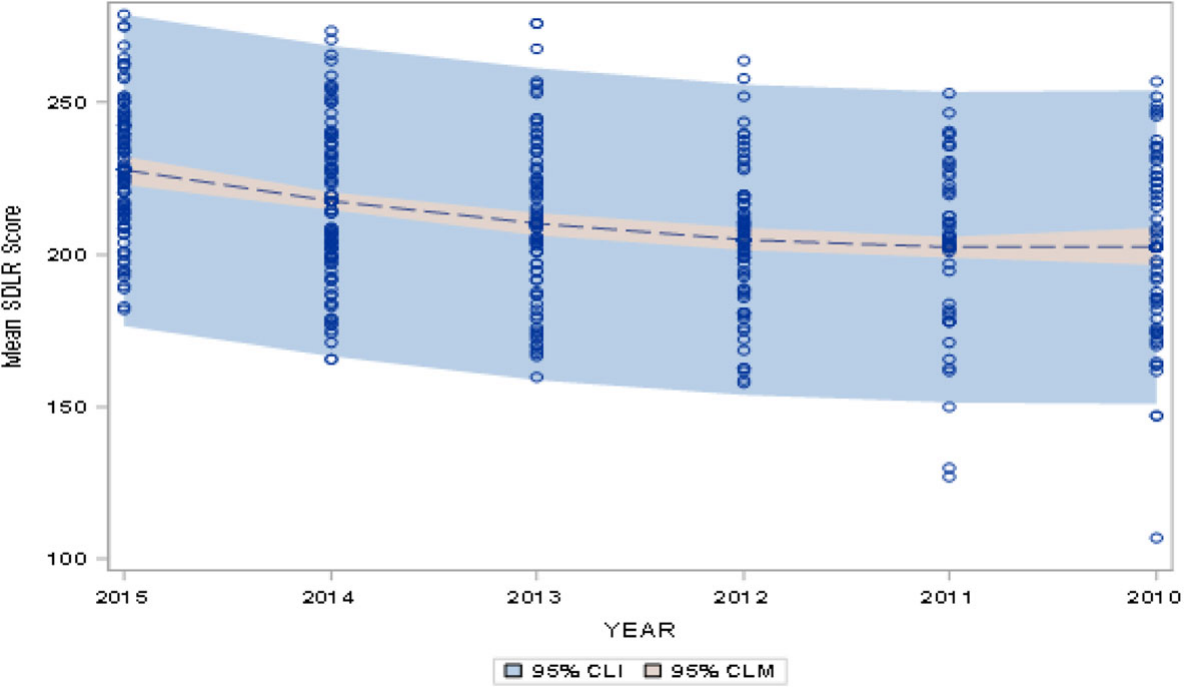
Fit Plot from regression model showing mean SDLR score at admission, end of 1st, 2nd, 3rd and 4th year of training, and at the beginning of Internship (Batch of 2015, 2014, 2013, 2012, 2011 & 2010 respectively based on year of admission)

All students were admitted into the college at about the same age (17–18 years). The overall mean SDLR score for medical students at CMC was 212.91 (Fig. 1). Data showed that there was a significant drop in SDLR scores (*p* < 0.01) on comparing students at admission with those in medical training (Table 2; Figs. 2 and 3).

**Table 2:** Mean SDLRS scores of student cohorts

Cohort	n	Mean	SD	Lower 95%CL for Mean	Upper 95%CL for Mean
At admission	95	228.81	21.88	224.35	233.27
End of 1st Year	95	215.87	25.89	210.60	221.15
End of 2nd Year	79	211.64	27.23	205.50	217.78
End of 3rd Year	75	205.08	23.10	199.77	210.40
End of 4th Year	53	203.45	28.11	195.71	211.20
Beginning of Internship	56	202.11	31.86	193.57	210.64

Interpretation of scores: Low (58–176); below average (177–201); average (202–226); above average (227–251); high (252–290)

The speed of decline appeared to decrease at latter time points (Figs. 2 and 3).

### Qualitative analysis

Qualitative data captured perceptions on the role of culture and the curriculum on SDL from 5 student focus groups and 7 faculty interviews.

### Perceptions of SDL

#### Definitions

There was general working knowledge of SDL across the cohorts. The definition was more specific with students in more advanced years of study highlighting the key elements of SDL in their definitions compared to earlier years:

*‘SDL is something that you can learn from, something that is different, you are usually motivated to go back and read up more about the subject and is not limited to what your teacher tells you in that half an hour, or one hour’ (Batch 2011, end of final year).*

*‘It should also include, understanding your own potential, how much you can learn, you should learn, basically it is like time management’ (Batch 2014, end of first year).*

*‘Understanding your own skill and abilities and learning in a specific pattern that adapts to that’ (Batch 2015, at admission).*

Faculty expressed their understanding of SDL in multiple perspectives. Some faculty were of the opinion that SDL should be initiated by instructors while others noted that the student takes sole ownership of the learning process, as expressed in the different definitions given:

*‘SDL,...have a small discussion at the start of the topic and certain areas we can cover and then give certain areas they can go and learn and then come back and have a discussion rather than direct lectures, and if it is done in small groups it will be a better way’ (F3).*

*‘Usually I would like students in India to get into this form of SDL which according to me is students are able to gather, secure information on their own and also internally motivate to learn on their own and have a desire for learning on their own cause ….In my definition, what it would encompass would be internal motivation, looking for resources on their own and having a desire to learn not being pushed by anybody, the teacher or the parents’ (F4).*

#### Activities in curriculum and other factors that promote SDL

Students indicated that when faculty explained a topic very well and promoted interactive sessions in class by asking questions, this motivated SDL because the students sought to read more in order to close the knowledge gap identified. Similarly, if the faculty presented a new clinical case or situation that is different, the student is more self-directed in studying. The Integrated Learning Program (ILP) [9] was repeatedly noted by both students and faculty to promote SDL. As indicated in this statement:

*‘ILP…. it was an integrated program for the three subjects. We had a lot of experience there and gathered more knowledge and that is when we realized that we should go and read other books’* (Batch 2014).

Students in the advanced years of study identified practical case discussions, observation of doctors in clinical settings and clerkship (where students are posted in the clinical wards and are involved in patient care and management from admission to discharge) as key features of the curriculum that promotes SDL because of the different mode of learning:

*‘SDL affects us more during clerkship - the clinical posting. We are fully involved in how the patient is managed which is different from how we get to know in lectures, reading topics than the usual exam oriented clinics’* (Batch 2011).

Faculty indicated that clerkship in clinical years (2nd to 4th year), secondary hospital program, health education message development, eLearning modules, discussions on cases and clinical training were some learning activities in the curriculum that supported SDL. As some instructors stated:

*‘Chart discussion, we make some charts on cases and problems. They will be given time to read about it and find the answer themselves. Some mode of self-learning happens there. But again, ultimately, we discuss in detail. But at least, it encourages them to read by themselves’* (F1).

Faculty also believed that interactive classes via student led seminars, topic specific presentations by students, tutorials and providing students with lecture topics ahead of class promoted SDL. In addition, faculty perceived that pre-tests and post-tests, assessments given at the end of class and Objective Structured Clinical Examinations motivated students to be self-directed. Awards for excellence in studies were also a motivating factor for SDL.

#### Factors that deter SDL

Assessment was a key factor that both facilitated and deterred SDL. Some students perceived that assessment can drive SDL only if it means something to the final exams. However, others perceived assessments as a hindering factor to SDL. The curriculum is loaded with various activities targeted towards the various assessments which occur at frequent intervals of learning. Students are assessment-oriented and as such, all learning is focused on acquiring skills and knowledge that will enable them to excel in assessments. There is limited time set aside for SDL. Students considered that the frequency of tests was an SDL deterrent. In addition, faculty indicated that the current form of teaching is sometimes exam-oriented due to the rigid curriculum. This likewise influences instructors’ form of teaching in trying to achieve the required standard.

*‘Because the curriculum demands, I mean for all of us, we just want to get through the exams, so if you are studying something else, then you might be missing something that is important for your exams’* (Batch 2012).

*Each week there is an exam…so it is also a problem’* (F1).

*‘Regulations….They say that these are the topics that have to be covered, these are the exams, these are the marks, we are told very clearly this is the case, these are the exercises that we have to do, we have to concentrate to make sure that the students pass.’* (F3).

Other factors that deterred SDL at times included not being adequately questioned during clerkship or class, hence reducing motivation for SDL. Too much of extracurricular activities for some students takes up time that could be used for SDL. Faculty similarly noted that as a result of the curriculum demands and extracurricular activities, some students have insufficient time for SDL.

Also, faculty considered the age and state of maturity of students at admission to the college as a deterrent. Students are admitted into the college at a young age and so some students are not mature to adequately motivate themselves to be self-directed in their study:

*‘They get in at 17, and are out by 22 as doctors. Many of them are straight from high school. And therefore the expectation from us also is sometimes too much. We expect them to be,... they are playful, they are children, they want to have their fun also.’* (F2).

*‘… we must also look at our culture, traditionally, if you see, you know when our students come into the college, they are a little more like kids, they are just adolescents. Our students are still young, because they come in here 18, 17 years of age, so SDL to a large extent should have to be pushed by us, though it is called SDL.’* (F4).

Both faculty and students identified that the type of schooling/coaching before admission to medical college, learning background and environment of the student influences their way of studying. The way of learning which students are used to is based on the traditional curriculum of direct learning. Hence, the students expect to be ‘spoon–fed’, deterring SDL. Instructors also stated that distractions due to modern technology and excess socializing are SDL deterrents.

#### Culture and SDL

There were mixed perceptions on the impact of parents, culture and environment on education and SDL. Some students indicated that their parents motivated them to study by being supportive and continuously checking on their performance. Others were of the opinion that their parents were more interested if they pass their exams and not keen on their daily educational activities and gave the students freedom to make their own studying choices:

*‘Parents…..In school, they were like pushing us to study. Now that we are in college we tend to call them once or twice and then they tell us to study. But then I say, mamma I have things to do, they think we are under too much pressure, so they don’t stress us too much’* (Batch 2014).

On the other hand, the faculty play the role of parents in prompting students to study. Faculty emphasized the influence of culture and family on SDL. Faculty indicated that society and parents have a lot of influence on students’ learning. Parents motivate students to study in various ways including waking the students up in the morning to prepare for classes:

*‘I think the parents again have a lot of input into the Indian children’s life. I think we cannot get away from that and you know that is again part of our culture, so you see even to the extent of your mum still calling you up in the first year, waking you up for the eight o’clock class’ (F4).*

*‘So all parents want children to excel in their studies so actually, it is like, you go study in the morning, they pack their lunch, even the pencils in the box, sort of study, study, study’ (F6).*

Students attended special coaching classes to train for the entrance exams into medical college. Faculty and students both indicated that the preparation for the entrance exams is intensive and most students start attending coaching classes as early as in their 9th grade. A few participants perceived that as a result of the intense studying for admission exams, few first year students are ‘burnt-out’ and tired of studying on admission.

#### Suggestions to improve SDL

This study showed that students understand the importance of being self-directed, however constraining factors mentioned above limits them. Hence, key recommendations were primarily focused on self-management in respect to creating a balance of time and course workload, as well as guidance on SDL. Students indicated that faculty should make classes more engaging and interactive. They also required direction in readings and orientation on what to expect and how to learn:

*‘Personally I think in medicine, no one can teach you everything, you have to learn on your own but the difficulty comes in the fact that... we have been conditioned in a way since childhood, we were taught everything, and then suddenly, that is not what you need and we don’t know what to do, so if you need to make a change, actually that thing should happen then, because after 14 years and plus one year of this, it is kind of hard to change’* (Batch 2013).

In addition, students saw the value of using videos to teach and more practical sessions and earlier contact with patients.

Faculty concurred that curriculum should provide students with enough time to study. In addition, students need to be orientated on SDL and SDL should be tried in small groups first for better impact. They stated that peer learning and change in the assessments would also promote SDL.

*‘That is where the curriculum has to come in and you have to give enough time’ (F2).*

## Discussion

### Measurement of SDL

The study showed that there was a reduction in the SDLR in students across different curriculum years from admission year to the final year of studies. Again, the definition of SDL differed across the years with medical students in advanced years providing the key elements of SDL than in junior students thereby, showing varying understanding of SDL.

The medical students’ SDLR score indicate average self-direction. This study’s score was slightly lower than similar studies carried out in Hawaii, USA (Mean SDLR = 235.68) and Saskatchewan, Canada (Mean SDLR = 230.6) [1]. Nevertheless, the findings align with a similar longitudinal study [1] that found the students become less self-directed with medical training. Our findings were also similar to a study on SDLR of dental students which used the same SDLRS instrument [19].This indicates that there is a decrease in SDLR of medical students during training irrespective of the type of curriculum. For instance, the curriculum in Hawaii is problem-based while that of Sasktchewan is more traditional. The curriculum in the Indian medical school is different from both the others.

### SDLR and culture

Data shows the prominent role of culture and the learning environment on SDL readiness. Students and faculty identified similar curricular and cultural factors affecting SDL. Students are influenced by their culture and learning environment which subsequently sculpts their ability for SDL [20]. Differences in SDL readiness are strongly related to certain features of the country’s learning culture [17]. Medical students may either adopt deep learning, wherein they are motivated to study due to the interest in the subject or its professional application, or students may adopt surface learning, which associates with motivation to study as a result of the desire to complete a course or fear of failure [21]. The type of learning approach adopted, impacts the SDLR of medical students. The learning culture in India shows that some students do not actively seek information for themselves rather, they focus on learning with the goal to pass exams and achieve high grades (collectivism) [22]. Shah et al. [21] showed that at the completion of the first academic year, there is a progressive shift from deep to surface learning approach.

In India, students are also influenced to a greater extent by the power position of instructors. Thus, authority to impart information lies with the instructor who is considered to have superior knowledge and students are conditioned from childhood to accept what the instructor says rather than think for themselves [22]. The perception of power is a key obstacle in most Asian students which influences not only SDL readiness but also communication and learning strategies adopted [15]. Nevertheless, contextual factors such as a traditional, teacher-centred secondary education, and examination content not covered during learning sessions can inhibit or enhance medical students’ SDL [15].

Our results provide yet another perspective on SDL, particularly the impact of culture and the type of prior schooling students had before entering medical college in determining SDL readiness. The study by Choi and colleagues [23] shows that due to the transition from traditional high school education to PBL instruction, the students felt uncertain about their learning. Relationships between learning styles and student demographics exist [24] but, El-Gilany and Abusaad [2] noted that although most students had high levels of SDLR, there was no significant difference with socio-demographics of the students as well as their learning style.

### SDLR and gender

Studies on the relationship between SDLR and gender are variable. Our study findings align with another study with first year MBBS students in India which showed that there was no significant difference among male and female students [4]. Yuan et al. [25] also stated no difference in SDL readiness between male and female students.

Findings from a study of fifth year MBBS medical students in India showed that although there was a general low SDLR among the MBBS students, male students had higher scores than females [7]. In contrast, Cadorin et al. [26] found that females had higher SDLR scores.

### SDLR and age

Contrary to some western medical education such as in United States and Canada, obtaining a prior degree before admission into medical college is not a requirement in India. The age of admittance into the medical program in Indian colleges is 17–20 years. Students are young and with no prior higher education or work experience. The SDLR scores of the Indian medical students were slightly lower than that of students in western medical schools. Similar to medical students in the west, the SDLR scores dropped as they got older and advanced in years at the college. This was contrary to the findings of Klunklin et al. [27] where SDL readiness on nursing students, increased with age, maturity, and as students progressed across a course. Similarly, a SDLR study with Chinese nursing students showed that students in higher years of study had higher scores for SDLR than students in lower study years and postulates that maturity promotes developing self-directedness [25].

The students in this study are similar to the participants in a study conducted by Shankar et al. [28] in Nepal, wherein students were younger i.e. between 18 and 19 years as compared to medical students in the West; less independent, more dependent on family and teachers and less trained for SDL during their prior medical college school years. The study showed that scores improved at the end of first year of learning but significant improvement was seen only in self-management scores. On the other hand, another study revealed no significant influence of age but first year students demonstrated lower levels of SDLR [29]. These findings suggest that age and maturity is a determining factor of SDL.

### SDLR and curriculum

In line with previous work, our study showed that curriculum plays a major role in SDL and aligns with Towle and Cottrell [30] who indicated that SDL can be enhanced by providing students with explicit advance information about tasks, specific performance goals for assignments, rewards for task completion, flexible time that allows sufficient time for task completion, support for student learning such as personal tutors, feedback and appropriate summative assessment. Kohli and Dhaliwal [31] noted that mentoring of students by faculty and peers, might improve the learning environment for students. Inability of students to cope with academic workload deters SDL suggesting that the curriculum needs a re-look in respect to course content and delivery.

Our study indicates that students require support for SDL. Students need assistance to improve their self-management skills [32, 33] so as to take control over his or her own learning especially in respect to time, resources and learning strategies due to the packed curriculum. Various strategies for SDL can be strengthened so that students can improve on their SDL skills [34]. Saurabh and Agrawal [35] similarly stated that students require more case or problem-based studies, clinical orientations, innovative teaching programs group discussions and tutorials in regular teaching so as to improve their performance in exams and to make them more self-directed.

## Limitations of the study

A few participants seem to have misinterpreted some questions in the SDLR assessment tool, as revealed during the focus group discussions. Hence, although the instrument has been tested, validated and found reliable in other countries, the SDLR score of these students may not reflect the true score. In addition, data used are based on self-reports and students’ perceptions.

## Conclusion

Given the decline in SDLR between batches of students from admission year to the final year of studies and the importance of SDL in medicine, the current curriculum may require an increase in learning activities that promote SDL. This study points out the need to address medical students’ SDL skills and ways to build these skills. It also shows that curriculum, assessments and culture does impact SDL readiness.

Didactic lectures, tutorials, and practical classes are the common methods of teaching in most medical colleges of India [35]. In order to promote SDL, current teaching and learning strategies may need to be re-examined and modified. Faculty development plays an important role in implementing such changes.

The results of this study indicate that multiple factors play a role in SDLR of medical students. Further studies that monitor SDLR in postgraduate medical education can provide insight into how medical training transforms health professionals into lifelong learners.

## Additional file

Additional file 1: Interview guide. Contains questions that were asked during the focus group with students and interviews with faculty. (DOCX 13 kb)

## Abbreviations

ANOVA: Analysis of Variance
CMC: Christian Medical College
ILP: Integrated Learning Program
MCI: Medical Council of India
SDL: Self-directed learning
SDLRS: Self-directed Learning Readiness Scale
