# Supplementary material for: Self-directed learning readiness of Indian medical students: a mixed method study
Source: BMC Med Educ. 2018 Jun 8;18:134. doi: 10.1186/s12909-018-1244-9 (PMC5994133; doi:10.1186/s12909-018-1244-9)
Supplement: Supplementary file 1 — Interview guide. Contains questions that were asked during the focus group with students and interviews with faculty. (DOCX 13 kb) [file 12909_2018_1244_MOESM1_ESM.docx]

**Interview Guide**

- How would you define Self-directed Learning?

The literature definition of SDL is: 
Self-directed learning describes a process in which individuals take the initiative, with or without the help of others, in diagnosing their learning needs, formulating learning goals, identifying human and material resources for learning, choosing and implementing appropriate learning strategies, and evaluating learning outcomes. (Knowles, 1975, p. 18)

- Using this definition, what are the factors that promote SDL in your curriculum? Give examples.
- What are the factors that deter SDL? Give examples
- How do you think culture plays a role in SDL?
